# Postoperative Delirium in Patients with Oral Cancer: Is Intraoperative Fluid Administration a Neglected Risk Factor?

**DOI:** 10.3390/cancers14133176

**Published:** 2022-06-28

**Authors:** Katharina Theresa Obermeier, Moritz Kraus, Wenko Smolka, Jochen Henkel, Thomas Saller, Sven Otto, Paris Liokatis

**Affiliations:** 1 Department of Oral and Maxillofacial Surgery and Facial Plastic Surgery, University Hospital, LMU Munich, 80337 Munich, Germany; wenko.smolka@med.uni-muenchen.de (W.S.); sven.otto@med.uni-muenchen.de (S.O.); paris.liokatis@med.uni-muenchen.de (P.L.); 2 Musculoskeletal University Center Munich, Department of Orthopaedics and Trauma Surgery, University Hospital, LMU Munich, 80539 Munich, Germany; moritz.kraus@med.uni-muenchen.de; 3 Department of Anaesthesiology, University Hospital, LMU Munich, 81377 Munich, Germany; jochen.henkel@med.uni-muenchen.de (J.H.); thomas.saller@med.uni-muenchen.de (T.S.)

**Keywords:** head and neck surgery, oral cancer, postoperative delirium, fluid management, fluid balance

## Abstract

**Simple Summary:**

Squamous cell carcinoma is the most typical malignant tumor of the oral cavity (OSCC) and surgery, including tumor resection and neck dissection with an appropriate reconstruction, remains the first line of treatment. Postoperative complications delay the healing process, and can have negative consequences for the patient. This study aimed to evaluate the impact of intraoperative fluid administration on developing postoperative delirium, and to identify other parameters leading to an increased risk of delirium.

**Abstract:**

Squamous cell carcinoma (SCC) is a malignant tumor derived from squamous cells and can be found in different localizations. In the oral cavity especially, it represents the most common type of malignant tumor. First-line therapy for oral squamous cell carcinoma (OSCC) is surgery, including tumor resection, neck dissection, and maybe reconstruction. Although perioperative mortality is low, complications such as delirium are very common, and may have long-lasting consequences on the patient’s quality of life. This study examines if excessive fluid administration, among other parameters, is an aggravating factor for the development of postoperative delirium. A total of 198 patients were divided into groups concerning the reconstruction technique used: group A for primary wound closure or reconstruction with a local flap, and group B for microsurgical reconstruction. The patients with and without delirium in both groups were compared regarding intraoperative fluid administration, fluid balance, and other parameters, such as blood loss, duration of surgery and overall ventilation, alcohol consumption, and creatinine, albumin, natrium, and hematocrit levels. The logistic regression for group A shows that fluid intake (*p* = 0.02, OR = 5.27, 95% CI 1.27–21.8) and albumin levels (*p* = 0.036, OR = 0.22, CI 0.054–0.908) are independent predictors for the development of delirium. For group B, gender (*p* = 0.026, OR = 0.34, CI 0.133–0.879) with a protective effect for females, fluid intake (*p* = 0.003, OR = 3.975, CI 1.606–9.839), and duration of ventilation (*p* = 0.025, OR = 1.178, CI 1.021–1.359) are also independent predictors for delirium. An intake of more than 3000 mL for group A, and 4150 mL for group B, increases the risk of delirium by approximately five and four times, respectively. Fluid management should be considered carefully in patients with OSCC, in order to reduce the occurrence of postoperative delirium. Different factors may become significant for the development of delirium regarding different surgical procedures.

## 1. Introduction

Oral squamous cell carcinoma (OSCC) is a type of cancer arising from squamous cells in mucosal surfaces of the oral cavity, i.e., the area between the lips and the base of the tongue. The floor of the mouth, tongue, buccal mucosa, maxillary mucosa, the alveolar process, the vestibule, and lips are possible localizations. In particular, tobacco consumption, chronic alcohol abuse, and the human papillomavirus (HPV) are among the risk factors for developing OSCC [1]. Although new technologies are being implemented for the management of OSCC [2,3], the first-line treatment for this tumor entity remains, primarily, surgery, with or without chemoradiotherapy [4]. In addition to tumor resection, treatment could include neck dissection to remove the draining lymphovascular structures [5]. However, the most time-consuming part of the surgery is often the reconstruction of the bone or soft tissue defect resulting from the tumor resection. The reconstruction necessitates using local flaps or transplants, and microsurgical techniques to restore the anatomy and the essential functions performed in the oral cavity. Although the typical patient with OSCC suffers from many comorbidities, surgical treatment is generally well-tolerated, with a perioperative mortality of 0.1% [6]. Cardiopulmonary and respiratory complications are of particular interest, since they affect 10–15% of patients with OSCC, and are extensively discussed in the literature since they are the leading cause of perioperative mortality [7,8]. Although delirium commonly does not lead to increased perioperative mortality, it is a severe complication that can prolong hospitalization, increase costs, and, most importantly, reduce mental status, with permanent restrictions and a reduced quality of life for the patients [9,10].

Delirium is a multifactorial condition with different clinical presentations and a possibly varying pathophysiology. Many parameters were studied to identify surgical patients with an increased risk of developing this complication. Booka et al. (2016) report age > 70 years and a history of cognitive deficits as the main prognostic factors for developing postoperative delirium [11]. Shiiba et al. (2009) identify the male gender as a possible risk factor, and the application of intraoperative fentanyl as a protective factor. Crawford et al. (2020) find that excessive alcohol consumption causes a higher risk of postoperative delirium [12]. Further parameters, such as blood pressure, albumin levels, anesthesia drugs, perioperative hypotension, and hypoxemia, were also investigated as possible factors increasing the risk of delirium, with contradictory results [13,14,15]. Among many parameters studied, fluid balance is associated with delirium in patients with shock [16].

Adequate fluid administration without overloading the patient is often a challenge for the anesthesiologists, especially for multimorbid patients, where many factors must be taken into consideration. From a surgical perspective, limited fluid intake could lead to a lower risk of developing surgical or medical complications, such as flap loss, edema formation, and longer hospitalization [17,18,19]. On the other hand, Myles et al. (2018) describe a higher risk for kidney damage due to restrictive fluid management [20].

Although possible surgical complications occurring from a liberal fluid administration, such as flap failure, are studied in surgical patients with OSCC [17,18], there are, to the best of our knowledge, no studies investigating liberal fluid administration concerning the development of delirium in those patients. Moreover, in the existing literature, fluid intake is examined mainly in non-surgical patients as a predictor of the development of delirium [16,19]. For surgical patients with oral cancer, fluid intake is not investigated adequately, or separated from bleeding, to reveal its possible association with the development of delirium.

This study aimed to evaluate the impact of intraoperative fluid administration on developing postoperative delirium, and identify other parameters leading to an increased risk of delirium in surgical patients with oral cancer. Moreover, relevant confounders, such as bleeding, which could hide the association between fluid intake and delirium, were considered.

## 2. Material and Methods

This retrospective study was approved by the institutional Ethics Committee of the University Hospital of Munich, Germany (Munich, Germany; 20-1096). The patients treated in our department from 2014 to 2019 due to a diagnosis of an OSCC were retrospectively analyzed. Requirements for inclusion were curative treatment due to an OSCC with tumor resection, and uni- or bilateral neck dissection, and postoperative admission in to the intensive care unit. Exclusion criteria were moderate to severe, or not adequately treated, acute or chronic heart or kidney failure, and chronic liver dysfunction. Overall, 229 patients were treated due to the primary diagnosis of an OSCC between 2014 and 2019 in our department. Finally, 198 patients fulfilled the inclusion criteria and were included in the present retrospective study. Although the surgical treatment was performed due to the same diagnosis of an OSCC for all included patients, the decision to perform a microsurgical reconstruction was based on the extent of the tumor, and had a decisive impact on the intraoperative and postoperative surgical and anesthesiologic management of the patients. The patients were separated into two groups, according to the reconstruction performed, since this allows the comparison of homogenous groups and treatments, and can lead to more practical suggestions regarding fluid administration:

Group A: primary wound closure (PWC) or reconstruction with a local flap;
Group B: microsurgical reconstruction with a free flap.

For the diagnosis of delirium, the Nursing Delirium Screening Scale (Nu-DESC) was documented twice daily in the ICU. Patients who scored ≥ 2 points in at least one evaluation were classified as delirious. The patients who suffered from postoperative delirium, as documented in the intensive care unit, were identified and compared to those without delirium, in order to identify relevant factors.

### 2.1. Outcome Measures

Demographic, medical, and oncological data were collected for all patients. The outcome parameter examined was the presence or lack of delirium in the days following the extubation, or the end of ventilation in tracheotomized patients.

The primary variable examined regarding the development of delirium was intraoperative fluid administration during surgery. In addition, fluid balance was calculated by adding intraoperative blood and urine loss, and subtracting it from administered fluid during surgery. Further parameters evaluated were age and gender of the patients, pre-existing dementia, duration of surgery, duration of intubation, and alcohol consumption (cut-off: 21 units/week). The laboratory parameters evaluated were total protein, albumin, hematocrit, natrium, and creatinine levels.

In order to identify which of the factors mentioned above could be associated with the development of postoperative delirium in our patient population, we performed a comparative exploratory data analysis, in which we compared the patients with a postoperative delirium with those without it, and tested the parameters mentioned above for statistically significant differences.

### 2.2. Statistical Analysis

Statistical analysis was conducted using the software SPSS Statistics 26 (IBM, Armonk, New York, NY, USA).

Firstly, we defined cut-off values, implementing the ROC analysis for groups A and B separately for the quantity of administered fluid leading to an increased risk of delirium. The fluid administration was examined as a categorical variable regarding these cut-off points.

Followingly, the data were tested for normal distribution with the Shapiro-Wilk-Test. The *t*-test and Mann–Whitney–U test were selected for hypothesis testing for parametric and non-parametric continuous data, respectively. For nominal data, the x^2^ test was used. The significance level was defined at *p* ≤ 0.05. Where necessary, alpha adjustment, according to Benjamini–Hochberg, was performed for multiple testing.

The parameters that were significantly associated with the development of delirium in the univariate analysis were further investigated, using a binomial multivariate logistic regression to determine associations between the selected co-variables and the occurrence of delirium. The Pearson and Spearman correlation tests were used to check for multicollinearity between theoretically associated variables (i.e., fluid balance, fluid administration, and blood loss). Since it is not appropriate to examine collinear variables in the same multivariate regression model, logistic regression with forward selection was used to decide which collinear variable is better associated with the development of delirium and should be included in the multivariate regression model. The logistic regression results were summarized with odds ratios and 95% confidence intervals (CIs). Model calibration was summarized using the Hosmer–Lemeshow goodness test.

## 3. Results

Oncological characteristics and details about the performed surgical procedures for groups A and B are shown in Table 1.

### 3.1. Results Group A

Overall, 98 patients (39 female, 59 male) are included in group A. The mean age is 65.4 (30–89 years). The median surgery duration is 300 min (150–660 min). The median fluid administration during surgery amounts to 3000 mL (500–6500 mL). Median blood loss during surgery amounts to 300 mL (100–1800 mL), median loss due to urine is 725 mL (100–4000 mL). The median fluid balance is +1700 mL (range from −1390 to +4450 mL). The median sedation time in the ICU is 2 days (0–25 days), while the medium stay in the ICU is 4 days (2–32 days). A total of 23.5% (n = 23) of the patients report regular consumption of alcohol, while eight patients have a history of dementia. The median albumin value is 3.3 g/dL (2.1–5.3 g/dL).

The cut-off value for the fluid intake estimated with the ROC curve is 3000 mL.

Overall, 18 patients (18.2%) suffer from delirium in group A. Of these patients, 5 received fluids below 3000 mL, and 13 more than 3000 mL.

**Figure 1:**
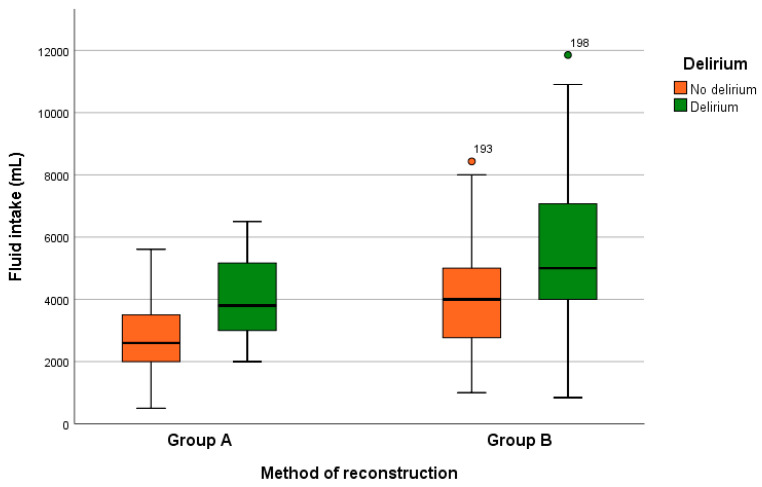
Occurrence of delirium for different surgical procedures concerning fluid intake. A total of 18.2% of the patients in group A and 48% of the patients in group B develop delirium. (Group A: reconstruction with primary wound closure or local flap. Group B: microsurgical reconstruction with free flap).

The univariate analysis with the Mann–Whitney–U and x^2^ tests shows a statistically significant correlation between the development of delirium and fluid intake (*p* = 0.004), blood loss (*p* = 0.003), duration of ventilation (*p* = 0.019), duration of surgery (*p* = 0.036), and albumin levels (*p* = 0.039) (Figure 1). The co-variables that are not statistically significant for the development of delirium in our cohort are shown in Table 2.

All statistically significant parameters are investigated with the multivariate analysis. The data are tested for possible collinearity, and a strong correlation is found between blood loss and fluid administration, but not for fluid balance. Since strongly correlated variables cannot be examined in the same multivariate model, a forward selection analysis is selected to choose the variable statistically more significantly associated with the development of delirium between fluid intake and blood loss (Table 3). Fluid intake is more strongly associated, and can better predict the development of delirium than blood loss, so it is included in the multivariate model. The logistic regression shows that fluid intake (*p* = 0.02, OR = 5.27, 95% CI 1.27–21.8) and albumin levels (*p* = 0.036, OR = 0.22, CI 0.054–0.908) are independent predictors for the development of delirium. The Hosmer–Lemeshow test shows that our model has a good fit to the data (*p* = 0.137).

### 3.2. Results Group B

Overall, 98 patients (37 women, 61 men) are included in group B. The mean age is 64.6 years (32–88 years).

The median surgery duration is 456 min (260–780 min). The median fluid administration during surgery amounts to 4500 mL (840–11,850 mL). Median blood loss during surgery amounts to 500 mL (200–2500 mL), median loss due to urine is 900 mL (200–2500 mL). The median fluid balance is +2750 mL (range from −900 to +9150 mL). The median sedation time in the ICU is 5 days (1–30 days), while the medium stay in the ICU is 8 days (2–56 days). A total of 38.1% (n = 37) of the patients report regular consumption of alcohol, while seven patients have a history of dementia. The median albumin value is 3.1 g/dL (1.6–5.3 g/dL).

The cut-off value estimated from the ROC curve is 4150 mL.

Overall, 47 patients (48%) suffer from delirium in group B. Of these patients, 14 received fluid less than 4150 mL, and 33 more than that.

The univariate analysis shows a statistically significant correlation between the development of delirium and fluid intake (*p* = 0.001), blood loss (*p* = 0.00), gender (*p* = 0.006), and duration of the ventilation (*p* = 0.001) (Figure 1). Other examined factors are not statistically significant for the development of delirium.

The Pearson’s and Spearman’s tests reveal, as expected, collinearity between fluid intake and blood loss. According to that finding, and after implementing the forward selection analysis, which shows that fluid intake can replace blood loss for the prognosis of the development of delirium, the fluid intake is included in the multivariate model in addition to the other parameters identified from the univariate analysis as statistically significant. The logistic regression identifies gender (*p* = 0.026, OR = 0.34, CI 0.133–0.879) with a protective effect for females, fluid intake (*p* = 0.003, OR = 3.975, CI 1.606–9.839), and duration of intubation (*p* = 0.025, OR = 1.178, CI 1.021–1.359) as independent predictors for the development of delirium. The Hosmer–Lemeshow test shows that our model has a good fit to the data (*p* = 0.44).

## 4. Discussion

Delirium is a multifactorial condition, and although many attempts to identify risk factors for surgical patients were made, the etiology remains unclear, and there are no common strategies to prevent it. Delirium is reported to affect up to 73.5% of the patients after severe operations [21]. According to its manifestations, it is classified into three types: hyperactive (patient is agitated, possibly with hallucinations), hypoactive (patient is disorientated, sleepy, and inactive), and mixed [22].

In head and neck surgery, delirium affects 17 to 33.3% of patients [23,24,25,26]. Surgical procedures that include tumor resection, neck dissection, and reconstruction with microsurgical tissue transfer are associated with higher delirium rates than procedures where no flaps or pedicled flaps are used for reconstruction [27]. In our study, the overall occurrence of delirium is 33.2%, in accordance with the literature. However, when the patients with and without free flaps are separately examined, the rates are 18.2% and 48%, respectively. There is a statistically significant difference between the patients with or without free flap reconstruction (*p* = 0.003). The delirium rate among the patients who receive a free flap reconstruction is higher than that reported in the literature [25]. This discrepancy could be explained by the longer ventilation time in our patients compared to those of Makiguchi et al. [25], and the different criteria used for diagnosing delirium, since the study of Makiguchi et al. uses the criteria according to DSM, which may have different sensitivity and specificity compared to the Nursing Delirium Screening Scale (Nu-DESC) implemented in our study [28,29].

In previous studies, many prognostic factors are examined regarding delirium in patients with OSCC. In these studies, the following factors are identified as independent predictors for the development of delirium: older age (>70 years) [11,27,30], lower intraoperative Hb levels [30], excessive bleeding during surgery with transfusions [30,31], operation time more than 7 to 10 h [24], high preoperative albumin values [25], postoperative insomnia [25], history of smoking [25], excessive alcohol consumption [12], time to ambulation after surgery [27], gender [32], postoperative pain control [32], and sedation period [26]. Excessive blood loss requiring transfusions is a commonly accepted factor that increases delirium risk, and is controlled in many study protocols. On the contrary, although excessive crystalloid fluid intake is thought to impact the development of delirium in patients with other conditions who are not bleeding [16], this is only scarcely studied in patients with OSCC, and no statistically significant influence is found in the multivariate analyses [24,32]. A reason for that could be the collinearity of the two co-variables, since increased bleeding leads to increased fluid administration, which could hide the impact of excessive positive fluid balance if the statistical analysis does not consider that, especially if the sample is relatively small.

In our cohort, the same parameters were analyzed separately regarding the surgical procedure performed on the patients. Fluid intake is an independent risk factor in both groups. Moreover, multivariate analysis reveals that in group A, low albumin and in group B, male gender and sedation duration could influence the occurrence of delirium. The volatility regarding the predictors for delirium between studies, and even in the present study between groups, could result from the multifactorial etiology of delirium. In group A, the median sedation duration is two days and is not an independent predictor, while in group B, it is five days and statistically significant for delirium. This indicates that the sedation time could only play a significant role if it exceeds a certain duration, otherwise, it could be replaced as a predictor with other, more decisive parameters, such as fluid intake, which express the extent of the surgical procedure or the preoperative condition of the patient. This volatility of the predictors for the same diagnosis but a different procedure is an interesting finding of our study, and could also explain the differences between other studies. It also highlights the importance of fluid intake in developing delirium, since it can replace other predictors if it exceeds a certain amount.

In our data, alcohol abuse is not statistically significant. Overall, only 23.5% (n = 23) of the patients in group A and 38.1% (n = 37) in group B report regular consumption of alcohol. The actual number could be higher. However, alcohol abuse could be a possible confounder, and can be accurately evaluated only in prospective studies, due to the high percentage of patients not reporting it [33].

Another finding of this study is the excessive positive fluid balance in our patients’ cohort. The median fluid balance is +1700 mL for group A, and +2750 mL for group B. Although this is desired in some conditions, for example in shock patients, it could lead to complications in the surgical patient, and it should be seen with caution by the anesthesiologist.

Furthermore, our results show that an intake of more than 3000 mL for relatively minor operations (group A) and 4150 mL for relatively major procedures operations (group B) increases the risk of delirium by approximately five and four times, respectively. Other studies regarding patients with OSCC propose 5500 mL as a cut-off point for an increased rate of surgical complications [17], and 5000 mL for an increased rate of delirium [34]. In conclusion, a fluid volume of more than four to five liters of crystalloid fluid correlates with an increased risk of complications.

The findings of this study indicate that possible measures to decrease the occurrence of delirium are a decrease in the postoperative sedation period and intraoperative fluid intake, wherever this is possible. Regarding the prolonged postoperative intubation and sedation period, which may be the wish of some surgical teams, especially in patients with free flap reconstructions, a prospective study from Nkenke et al. shows that, in patients who have relatively few comorbidities, the stay in the ICU does not reduce complications [34]. In compliance with these findings, another prospective study from Takahashi et al., on patients with few comorbidities and younger than 75 years, shows that early ambulation significantly decreases the occurrence of delirium [27].

Moreover, although bleeding is a significant parameter in our study, it could be replaced by fluid intake as a prognostic factor for the occurrence of delirium. In practice, we can take it as a given that the surgeon aims to minimalize bleeding, and as a consequence, this parameter can barely be improved to avoid delirium. On the other hand, according to our study, fluid management may be a parameter where a more restrictive administration could further reduce the risk of developing postoperative delirium.

Furthermore, although bleeding is a statistically significant parameter in our study, it could be replaced by fluid intake, which is a better predictor of delirium. In practice, we can take it for granted that the surgeon seeks to minimize bleeding and, consequently, this parameter can be minimally improved to avoid delirium. On the other hand, according to our study, fluid management may be a parameter where a more restrictive administration could further reduce the risk of postoperative delirium.

A disadvantage of the study is its retrospective character, which does not allow a targeted questioning of factors that could be important confounders for developing delirium, such as smoking and alcohol consumption. Moreover, the absence of a control group is a limiting factor, too. Additionally, due to the multifactorial etiology of delirium, some parameters that could play a role in the development of delirium may not have been considered in this study. However, these drawbacks could be partially compensated by the detailed documentation of the patients’ management, complications, and the course that takes place in the intensive care unit. Last, but not least, a strength of the study is the relatively high number of patients included, and the classification of the patients into two groups, which allows the investigation of similar surgical and anesthesiologic procedures, resulting in more practical guidance regarding fluid administration.

## Figures and Tables

**Table 1:** Overview of demographic and oncological data for groups A and B.

	**Group A**		**Group B**	
	**No Delirium**	**Delirium**	**No Delirium**	**Delirium**
**Median age (years)**	65.1	66.5	64.9	64.2
**Gender**				
Male	34	6	36	25
Female	48	12	11	26
**Tumor Localization**				
Mouth floor	24	8	13	23
Tongue	31	2	23	8
Planum bu.	3	1	8	2
Maxilla	10	3	2	4
Lip	6	2	1	0
Alveolar	7	2	5	10
**T stadium**				
T1	47	6	10	5
T2	27	5	19	16
T3	1	2	9	4
T4	6	5	12	18
**N stadium**				
N0	59	10	31	21
N1	11	2	2	5
N2a	1	1	0	4
N2b	3	3	7	9
N2c	0	1	2	5
N3b	-	-	5	2
**Free flap**				
RFF			36	27
Scapular			5	9
Iliac crest			8	7
Fibula			3	4
**Neck dissection**				
One-sided	17	6	12	5
Both-sided	65	12	40	42
**Alcohol abuse**	16	7	17	20
**Dementia**	6	2	3	0

**Table 2:** Co-variables and *p*-values in the univariate analysis for groups A and B.

Parameters Examined	Group A		Group B	
	Median (Range)	*p*-Value	Median (Range)	*p*-Value
Creatinine	0.8 µmol/L (0.3–1.8)	0.296	0.8 µmol/L (0.3–3.6)	0.672
Total protein	6.6 g/dL (4.1–12.4)	0.816	6.5 g/dL (3.3–8.1)	0.803
Urine loss	675 mL (0–4000)	0.092	900 mL (200–2500)	0.250
Natrium	145 mmol/L (130–149)	0.42	143 mmol/L (132–150)	0.478
Hematocrit	0.355 L/L (0.288–0.475)	0.94	0.299 L/L (0.184–0.407)	0.736
Blood loss	300 mL (50–1800)	**0.03**	500 mL (50–2100)	**0.00**
Surgery duration	300 min (150–660)	**0.036**	456 min (260–780)	0.303
Duration of ventilation	2 days (1–25)	**0.019**	5 days (1–30)	**0.001**
Age	65 years (30–89)	0.561	64 years (32–88)	0.53
Gender		0.267		**0.007**
Preexisting dementia		0.604		0.801
Fluid intake	3000 mL (50–6500)	**0.004**	4500 mL (843–11,852)	**0.001**
Albumin	3.3 g/dL (2.5–5.3)	**0.039**	3.1 g/dL (1.6–5.3)	0.376
Alcohol consumption		0.084		0.314

**Table 3:** Co-variables in the regression analysis for groups A and B.

Parameters	Group A			Group B		
	*p*-Value	Odds Ratio	95% CI	*p*-Value	Odds Ratio	95% CI
Duration of ventilation				0.031	1.17	1.02–1.35
Gender				0.043	2.69	1.03–6.99
Fluid intake	0.02	5.27	1.27–21.8	0.003	3.975	1.61–9.84
Albumin	0.026	0.2	0.48–0.28			

## Data Availability

The data presented in this study are available on request from the corresponding author.
